# The De La Rue Scheme of Medical Service

**Published:** 1920-07-24

**Authors:** Stuart De La Rue

**Affiliations:** 110 Bunhill Row, E.C. 1.


*To the Editor of* The Hospital.

### The De La Rue Scheme of Medical Service.

### THE FIRM'S TRUE INTENTIONS.

*To the Editor of* The Hospital.

Sir,—I have read with interest your criticisms of my company's scheme of medical service, and would ask you to allow me to correct certain misapprehensions as to its nature and its scope which have arisen by my desire to describe so complex a question in so short a memorandum as was sent to you.

(1) The patients have the choice in the matter of consultants, and there are at least two consultants to each department. They can by going to one of these have the benefit of the reduced fees arranged for by the company, but they are at perfect liberty to go to any other consultant of their choice, and either pay his full fees or such fee as they may themselves arrange. Nor are the patients in any way compelled to visit the company's doctor, so that they retain in the fullest measure freedom of choice in the selection of their advisers.

(2) The general practitioner is not, as you suppose, ignored—indeed, he works in consultation with the specialists; but to protect the profession against an abuse of the concessions they have so generously made a provision is inserted that the certificate of the company's doctor is required, who, having given it, drops out of the case altogether. In most cases this would be given on an application in writing from the general practitioner to the company's doctor, who would not require or need to see the patient.

(3) The large nursing home idea is in the embryonic state. It is obvious that such a home could be run more efficiently and cheaply than the small homes now patronised. It is also obvious that large employers would be glad of the provision of such facilities at low rates for their people, and might be ready to support their desire with financial capital assistance. It would be no part of the scheme to exclude the general practitioner, and probably no part to attach only a limited number of consultants to the home, but it would probably be part of the scheme that any consultant who would conform to the scale of reductions agreed upon between the managers of the home and the medical profession should be free to make use of the facilities which such a home would provide.

In conclusion, I would like to say how much your kind remarks about our intentions are appreciated, and I truly regret that I did not make the whole proposal more clear to you in the first instance.—Yours very truly,

Stuart de la Rue.

110 Bunhill Row, E.C. 1.

19th July, 1920.

